# Spectral CT parameters of perivascular adipose tissue as non-invasive biomarkers for identifying symptomatic carotid atherosclerosis

**DOI:** 10.1186/s13244-026-02354-w

**Published:** 2026-07-22

**Authors:** Xiaohan Zheng, Xuehuan Liu, Jinyu Song, Endong Zhao, Weiwei Cui, Gouling Zhan, Zuoxi Li, Hong Yu, Xiao Gao, Jun Liu

**Affiliations:** 1 Tianjin Fourth Central Hospital, The Affiliated Hospital of Tianjin Medical University, Tianjin, China; 2 Department of Radiology, Tianjin Union Medical Center, Tianjin, China; 3 Medical School, Faculty of Medicine, Tianjin University, Tianjin, China

**Keywords:** Carotid atherosclerosis, Computed tomography angiography, Perivascular adipose tissue, Spectral computed tomography, Symptomatic carotid plaque

## Abstract

**Objectives:**

To evaluate the association between computed tomography (CT) spectral parameters of perivascular adipose tissue (PVAT) and carotid plaque composition, and to assess their value for identifying symptomatic plaques.

**Materials and methods:**

In this study, 306 consecutive patients with computed tomography angiography (CTA)-confirmed carotid atherosclerosis who underwent head and neck spectral CT angiography were analyzed. Quantitative plaque parameters and PVAT spectral metrics were extracted. Correlations between quantitative plaque parameters and PVAT spectral indices were analyzed, and logistic regression was used to identify independent predictors of symptomatic plaques. Diagnostic performance was evaluated by generating receiver operating characteristic (ROC) curves.

**Results:**

Compared with asymptomatic patients, symptomatic carotid plaques showed higher PVAT effective atomic number (*Z*eff) and iodine concentration (IC) but lower fat fraction (FF) (all *p* < 0.05). *Z*eff and IC correlated positively with fibrous fatty and necrotic core volumes, whereas FF correlated negatively with necrotic core volume (all *p* < 0.001). In multivariable models, *Z*eff, IC, FF, and virtual monoenergetic image attenuation at 70 keV (CT_70keV_) were independently associated with symptomatic plaques (all *p* < 0.05). CT_70keV_ and FF performed best for identifying symptomatic plaques (AUC 0.857 and 0.820). The AUC increased from 0.716 for stenosis severity alone to 0.821 after the addition of necrotic core volume, and further to 0.897 and 0.916 after the addition of FF and CT_70keV_, respectively.

**Conclusion:**

Spectral CT-derived PVAT parameters differ between symptomatic and asymptomatic carotid atherosclerosis and are associated with plaque composition, providing complementary noninvasive information for identifying symptomatic plaques.

**Key Points:**

- **Question:** Can spectral CT-derived PVAT parameters improve noninvasive identification of symptomatic carotid atherosclerosis beyond luminal stenosis?
- **Findings:** PVAT spectral parameters differed between symptomatic and asymptomatic plaques, and adding FF or CT_70keV_ improved diagnostic performance.
- **Critical relevance statement:** spectral parameters of carotid PVAT differ significantly between symptomatic and asymptomatic carotid atherosclerosis and are associated with plaque components.

**Graphical Abstract:**

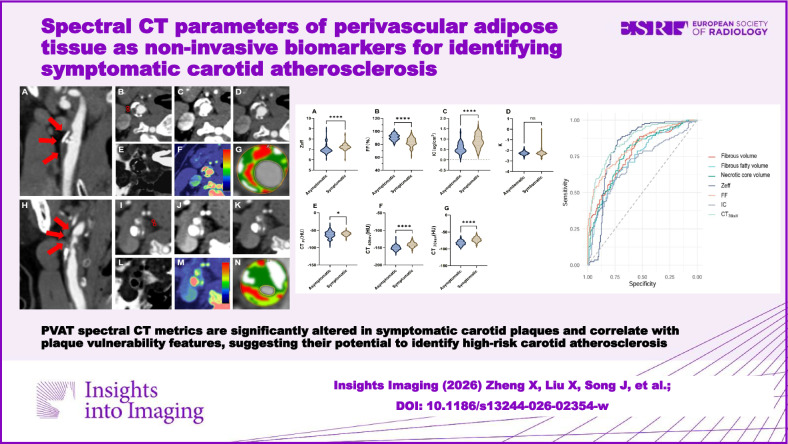

## Introduction

Carotid atherosclerosis is a leading substrate for ischemic stroke and transient ischemic attack (TIA), conditions that remain among the major causes of death and long-term disability worldwide [1]. Contemporary prevention and treatment strategies still rely largely on the severity of luminal stenosis [2]. There is growing evidence that carotid plaque vulnerability is associated with a higher risk of cerebrovascular events [3]. Features such as a large necrotic core, reduced fibrous content, and unstable surface morphology are strongly associated with downstream ischemic events [4], underscoring the need for imaging approaches that capture both structural and biological properties of carotid plaques rather than luminal narrowing in isolation.

Carotid atherosclerosis is a complex process that is not only influenced by plaque vulnerability but also associated with inflammation [5]. Perivascular adipose tissue (PVAT) is directly connected to the adventitia and plays a crucial role in regulating vascular tone, inflammation, and oxidative stress [6]. As an active organ with endocrine functions, PVAT undergoes changes in composition and biological properties due to inflammatory responses and engages in close bidirectional signaling with the vascular wall [7]. Several clinical studies have demonstrated the feasibility of non-invasive imaging techniques in measuring changes in PVAT [8–12]. These findings highlight the need for a more comprehensive stroke risk assessment that goes beyond the fragility of the plaque itself to include an assessment of the inflammatory environment.

Non-invasive imaging methods are increasingly used to characterize PVAT in vivo. While high-resolution magnetic resonance imaging (MRI) is considered the gold standard for assessing carotid plaque vulnerability, its clinical application is limited by long acquisition times, high cost, and reduced patient compliance [8]. Several CTA studies have reported that increased PVAT density around the carotid artery is associated with symptomatic stenosis, high-risk plaque features, and recurrent ischemic events, suggesting that PVAT density could serve as a surrogate of perivascular inflammation [13**–**15]. Nevertheless, most of these investigations quantified PVAT using a single mean attenuation value on conventional polyenergetic images (PIs), which only partially captures the complex compositional and functional changes within adipose tissue.

Spectral CT leverages the energy-dependent attenuation characteristics of X-rays to simultaneously reconstruct virtual single-energy images and substance-specific images, such as effective atomic number (*Z*eff) maps, iodine concentration (IC) maps, and fat fraction (FF) maps, thereby enhancing tissue composition discrimination [16]. These parameters provide complementary information across multiple dimensions, including tissue composition, blood supply status, and inflammatory state, and have been applied to assess carotid plaque composition [17, 18]. Therefore, these parameters may help refine PVAT phenotyping by capturing different aspects of tissue composition and contrast distribution beyond a single HU measurement. Additionally, studies suggest that PVAT spectral parameters may aid in identifying high-risk patients for acute stroke [19]. However, the relationship between PVAT spectral phenotypes and detailed plaque composition or clinical symptom status remains poorly elucidated.

Therefore, the aim of this study is to comprehensively analyze the relationship between PVAT and carotid plaque characteristics using spectral CT, and investigate the predictive value of these parameters in discriminating between symptomatic and asymptomatic plaques.

## Methods

### Patients

This retrospective study was approved by the Institutional Review Board, and informed consent was waived owing to its retrospective design. Patients suspected of carotid atherosclerotic disease who consecutively underwent spectral CTA at our institution from March 2025 to October 2025 were retrospectively enrolled. Eligible cases were identified by searching the institutional electronic medical record system and imaging database according to predefined criteria, and their clinical data and imaging information were systematically analyzed.

**Fig. 1:**
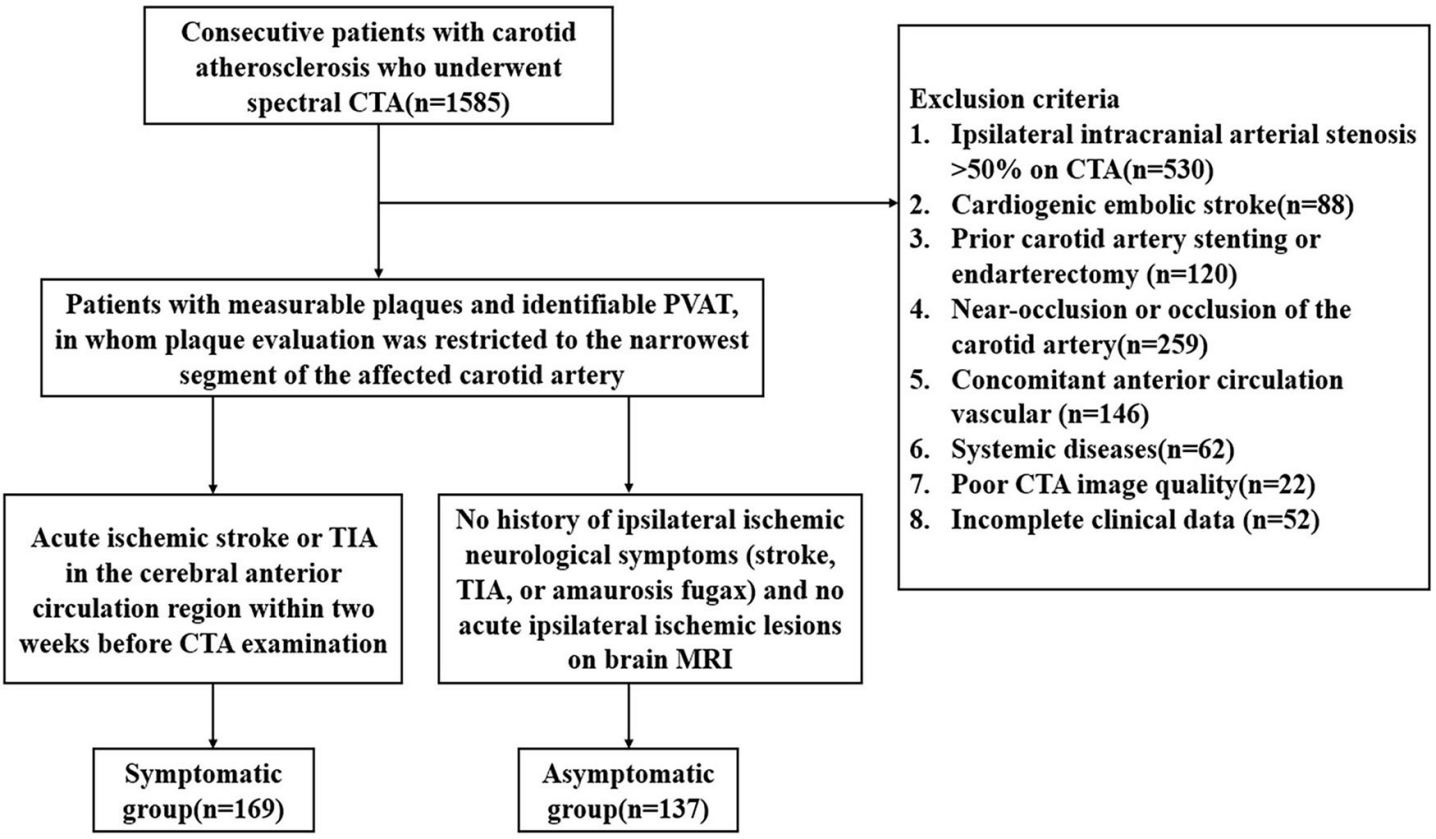
Flowchart of carotid CTA patients screened and assigned to study cohorts CTA, computed tomography angiography; PVAT, perivascular adipose tissue; TIA, transient ischemic attack; MRI, magnetic resonance imaging

The inclusion criteria were as follows: (1) age ≥ 18 years; (2) at least one carotid artery (unilateral or bilateral) with CTA-confirmed atherosclerotic plaque accompanied by measurable luminal stenosis; (3) clear identification and delineation of PVAT surrounding the target plaque; and (4) spectral CTA examination performed using the institution’s standard carotid scanning protocol. The exclusion criteria include: (1) ipsilateral intracranial arterial stenosis > 50% on CTA; (2) cardiogenic embolic stroke; (3) prior carotid artery stenting or endarterectomy; (4) near-occlusion or occlusion of the carotid artery; (5) concomitant anterior circulation vascular diseases (e.g., arterial dissection, aneurysm, or arteritis); (6) systemic diseases such as autoimmune disorders, hematological diseases, or malignancies; (7) poor CTA image quality precluding post-processing analysis; and (8) incomplete clinical data. The inclusion and exclusion process is illustrated in the flowchart (Fig. 1).

Patients with carotid plaques were considered symptomatic if they had ipsilateral ischemic stroke, TIA, or amaurosis fugax within 6 months before the CTA examination [18]. Brain MRI was used as supportive evidence to confirm ischemic lesions and their correspondence to the vascular territory supplied by the index carotid artery. Patients were classified as asymptomatic if they had CTA-confirmed carotid atherosclerosis without any documented history or imaging evidence of ipsilateral ischemic stroke, TIA, or amaurosis fugax within the preceding 6 months. To exclude prior TIAs in the asymptomatic group, we performed a structured medical-record review (admission notes, past medical history, discharge diagnoses, neurology consultation notes, and relevant prior brain imaging reports when available) to identify any prior ipsilateral ischemic events.

### CTA acquisition

All patients underwent head and neck CTA on a 256-slice GE Revolution Apex CT scanner (GE Healthcare) following a uniform protocol. The acquisition employed gemstone spectral imaging (GSI), a fast kV-switching dual-energy technique integrated with this scanner. Spiral scanning was used for CTA, with the coverage area extending from the aortic arch to the circle of Willis. A non-ionic contrast agent (Ioversol, 350 mg/mL) was administered via the elbow vein using a high-pressure syringe at a dose of 1.0 mL/kg body weight and a flow rate of 4.0 mL/s, a single injection dose is 50 mL. Scan parameters: the tube voltage was set with the GSI mode, ranging from 40 keV to 140 keV, tube current: 370 mA, matrix 512 × 512, reconstructed slice thickness 0.625 mm, helical pitch 0.992, slice interval 0.5 mm. The image sets included the conventional PIs and virtual monoenergetic image (VMI) sets from 40 to 140 keV.

### PVAT quantitative parameter analysis

The original images were transferred to Advantage Workstation 4.7 (GE Healthcare Revolution) for postprocessing. Two vascular radiologists (X.L. and J.S., with 15 and 10 years of experience, respectively) independently assessed and measured all plaque CTA characteristics using reconstructed images. During the image analysis process, the radiologists were blinded to all clinical data.

PVAT was measured on PIs and defined as adipose tissue located within a radial distance from the outer carotid artery wall equal to the vessel’s diameter, with attenuation values ranging from −190 to −30 HU [9]. We adopted a previously established method described by Baradaran et al [8], placing two regions of interest (ROIs), each measuring 2.5 mm², within the PVAT at the level of maximal luminal narrowing on CTA. The degree of carotid stenosis was calculated according to the North American Symptomatic Carotid Endarterectomy Trial (NASCET) method [20]. To minimize partial volume effects, each ROI was positioned at least 1 mm away from the outer boundary of the carotid artery and adjacent structures, carefully avoiding surrounding soft tissues and small penetrating vessels. The exact ROI locations varied among participants, depending on the anatomical position of the maximal stenosis and the distribution of perivascular fat. The mean value of the two ROIs was calculated and used for subsequent statistical analysis. Then synchronously replicate this ROI to the fat map, iodine map, *Z*eff map, and virtual monochromatic images. FF map derived from material-decomposition reconstruction (reported as %). IC was obtained from the vendor material-decomposition iodine map and was reported in μg/cm^3^. Based on prior spectral CT experience with PVAT imaging [21], we pre-selected 40 keV and 70 keV for quantitative PVAT assessment. Record CT attenuation of PVAT at conventional PIs, 40 keV and 70 keV monochromatic images (CT_PI_, CT_40 keV_, CT_70 keV_), plot the spectral attenuation curve, and determine its slope (K). K reflects the change in attenuation at a single energy level. Based on previous studies of spectral attenuation characteristics in adipose tissue [22, 23], the average attenuation of 40–70 keV VMIs shows a marked increase, then levels off above 70 keV. Therefore, we selected the 40–70 keV range to calculate the slope of the spectral attenuation curve. The formula is [23]: *K* = (CT_40keV_ – CT_70keV_)/30.

### Plaque quantitative features analysis

**Fig. 2:**
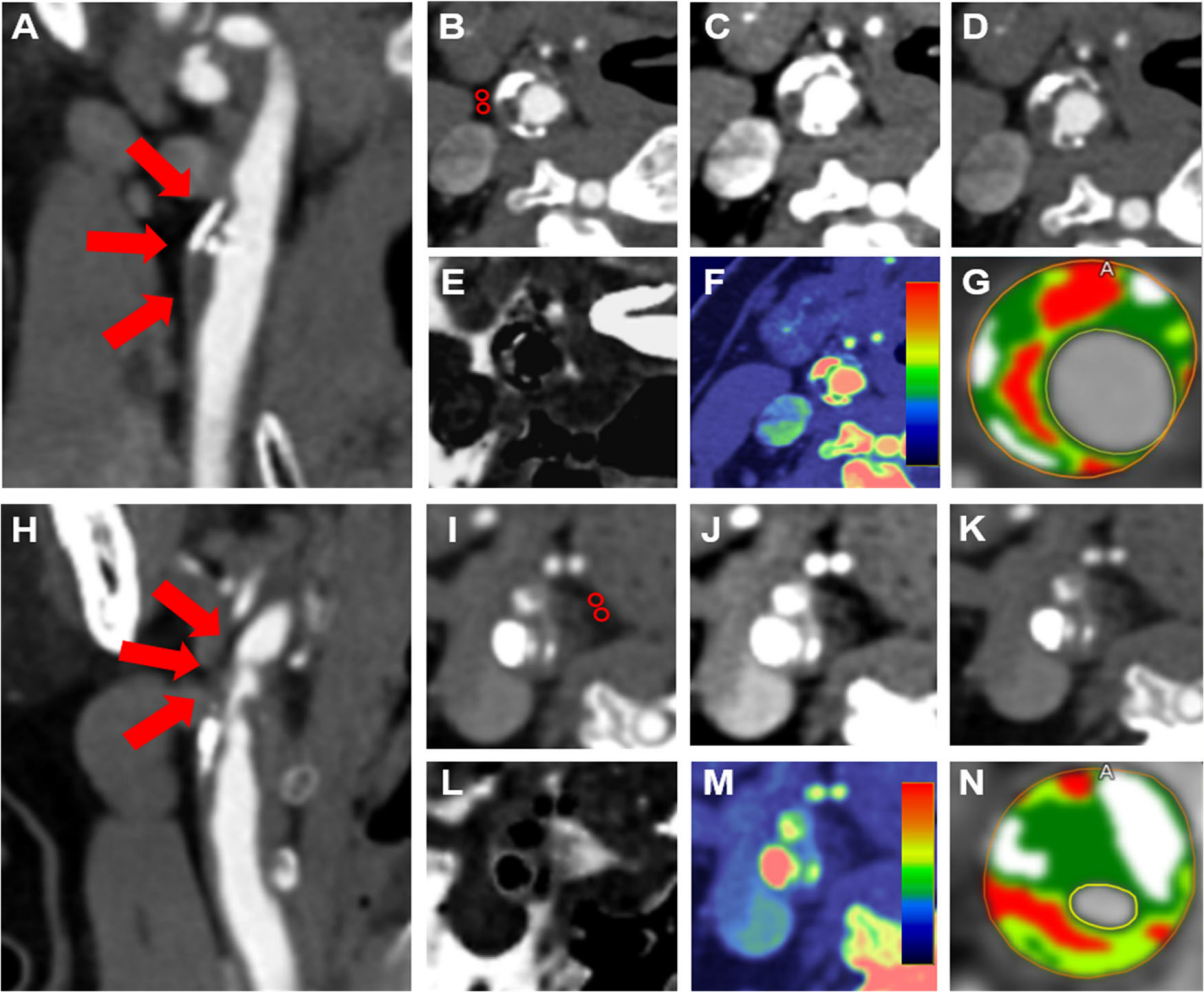
Representative comparison of PVAT spectral features and plaque composition in asymptomatic and symptomatic carotid plaques. Compared with the asymptomatic case, the symptomatic case shows relatively higher PVAT attenuation on conventional and VMIs, higher IC, and lower FF, together with plaque component features suggestive of increased vulnerability. **A** Asymptomatic carotid plaque (red arrow) in a 72-year-old man. **B** CT_PI_ = −58.64 HU. **C** CT_40keV_ = −162.20 HU. **D** CT_70keV_ = −78.70 HU. **E** Fat map showing FF = 92.79%. **F** Iodine map showing IC = 0.27 μg/cm³. **G** The software automatically quantifies volumetric plaque components, including fibrous volume (green) = 53.51 mm³, fibrous fatty volume (yellow-green) = 64.58 mm³, necrotic core volume (red) = 98.53 mm³, and calcium volume (white) = 60.89 mm³. **H** Symptomatic carotid plaque (red arrow) in a 71-year-old woman. **I** CT_PI_ = −57.03 HU. **J** CT_40keV_ = −141.40 HU. **K** CT_70keV_ = −72.20 HU. **L** FF = 79.91%. **M** IC = 1.58 μg/cm³. **N** Plaque composition analysis demonstrating fibrous volume (green) = 91.67 mm³, fibrous fatty volume (yellow green) = 106.38 mm³, necrotic core volume (red) = 88.73 mm³, and calcium volume (white) = 45.27 mm³

Vascular and plaque characteristics were assessed with a window width of 550 HU and a window level of 150 HU [11]. The degree of stenosis was evaluated using the NASCET criteria [20]. Plaque thickness: Maximum plaque thickness on axial CTA images [24]. Plaque length refers to the greatest distance measured along the plaque’s longitudinal axis. The data was then imported into Medis Suite software (QAngio CT) to extract quantitative parameters of carotid plaques, including plaque burden, fibrous volume, fibrous fatty volume, necrotic core volume, and calcium volume. Plaque component analysis was conducted utilizing the software’s predefined Hounsfield unit (HU) thresholds. Based on the built-in classification criteria, the attenuation values for each component are defined as follows: Dense calcification (≥ 350 HU), fibrous tissue (131–350 HU), fibrous fatty tissue (76–130 HU), necrotic core (−30 to 75 HU) (Fig. 2).

### Subgroup classification

To explore the potential effect of stenosis severity on PVAT characteristics and their predictive performance, patients were further stratified according to the severity of the index extracranial carotid artery stenosis based on the NASCET criteria: mild/moderate stenosis (< 70%) and severe stenosis (≥ 70%).

### Statistical analysis

All the statistical analyses were performed in SPSS for Macintosh, version 26.0 (IBM-SPSS) and R version 4.2.3. Data following a normal distribution were represented as mean ± standard deviation, while data not following a normal distribution were represented as median and interquartile range. Categorical variables were summarized as frequencies or percentages. Chi-square tests or Fisher’s exact tests were used to analyze differences in categorical variables between groups. For continuous variables, depending on the distribution, independent *t*-tests or Mann–Whitney *U*-tests were used for intergroup comparisons. Intraclass correlation coefficients (ICC) were calculated to assess the reliability and consistency of the imaging measurements for various carotid plaque features and PVAT parameters.

Spearman correlation analysis was employed to examine the relationship between carotid plaque CTA characteristics and spectral parameters of perivascular fat. Independent predictors of symptomatic carotid plaques were identified using univariate and multivariate logistic regression analyses, which were also used to develop a predictive model. Receiver operating characteristic (ROC) curves were generated using the predicted probabilities derived from the corresponding multivariable logistic regression models adjusted for clinical covariates, and area under the curve (AUC), sensitivity, and specificity were calculated. To evaluate the incremental discriminatory value of PVAT spectral parameters beyond stenosis severity and plaque composition, stepwise logistic regression models were constructed, and pairwise comparisons of AUCs were performed using the DeLong test. Bootstrap internal validation with 1000 resamples was further performed for the multivariable models, and bootstrap-based 95% confidence intervals were calculated for the regression coefficients (CIs) and odds ratios (ORs).

## Results

### Demographic and clinical characteristics

Three hundred six patients were included in this study, of which 137 (44.8%) were in the asymptomatic group, and 169 (55.2%) were in the symptomatic group. Demographic and clinical data are shown in Table 1. There was no statistically significant difference between the clinical characteristics of the two groups (*p* > 0.05).

**Table 1:** Demographic and clinical information of all participants

Variables	Total (*n* = 306)	Asymptomatic (*n* = 137)	Symptomatic (*n* = 169)	*χ*²/*t*/*Z*	*p*
Gender (male)	187 (61.11%)	83 (60.58%)	104 (61.54%)	0.03^a^	0.865
Age (years)	66.32 ± 11.08	65.69 ± 11.18	66.83 ± 11.01	0.90^b^	0.371
Hypertension	223 (72.88%)	92 (67.15%)	131 (77.51%)	3.60^a^	0.058
Diabetes	111 (36.27%)	53 (38.69%)	58 (34.32%)	0.45^a^	0.503
Coronary heart disease	92 (30.07%)	38 (27.74%)	54 (31.95%)	0.46^a^	0.500
TC (mmol/L)	4.58 (3.56, 5.26)	4.47 (3.66, 5.25)	4.71 (3.53, 5.26)	−0.67^c^	0.504
TG (mmol/L)	1.54 (1.19, 1.87)	1.50 (1.19, 1.80)	1.57 (1.19, 1.94)	1.06^c^	0.290
HDL-C (mmol/L)	0.97 (0.77,1.02)	0.90 (0.78, 0.95)	1.03 (0.77, 1.02)	1.24^c^	0.216
LDL-C (mmol/L)	2.57 (2.02,3.12)	2.53 (2.02, 3.12)	2.62 (1.99, 3.20)	−0.88^c^	0.378
Smoking history	116 (37.91%)	66 (48.18%)	50 (29.59%)	0.12^a^	0.734
Drinking history	61 (19.93%)	24 (17.52%)	37 (21.90%)	0.65^a^	0.419
Antihypertension use	217 (70.91%)	95 (69.34%)	122 (72.19%)	0.18^a^	0.676
Statin use	194 (63.40%)	86 (62.77%)	108 (63.91%)	0.01^a^	0.932
Antiplatelet use	71 (23.20%)	32 (23.36%)	39 (23.08%)	3.34^a^	0.068

*TC* total cholesterol, *TG* triglycerides, *HDL-C* high-density lipoprotein cholesterol, *LDL-C* low-density lipoprotein cholesterol

^a^ The statistical value is χ²

^b^ The statistical value is *t*

^c^ The statistical value is *Z*

### Differences in carotid plaque characteristics and PVAT spectral parameters between symptomatic and asymptomatic groups

**Fig. 3:**
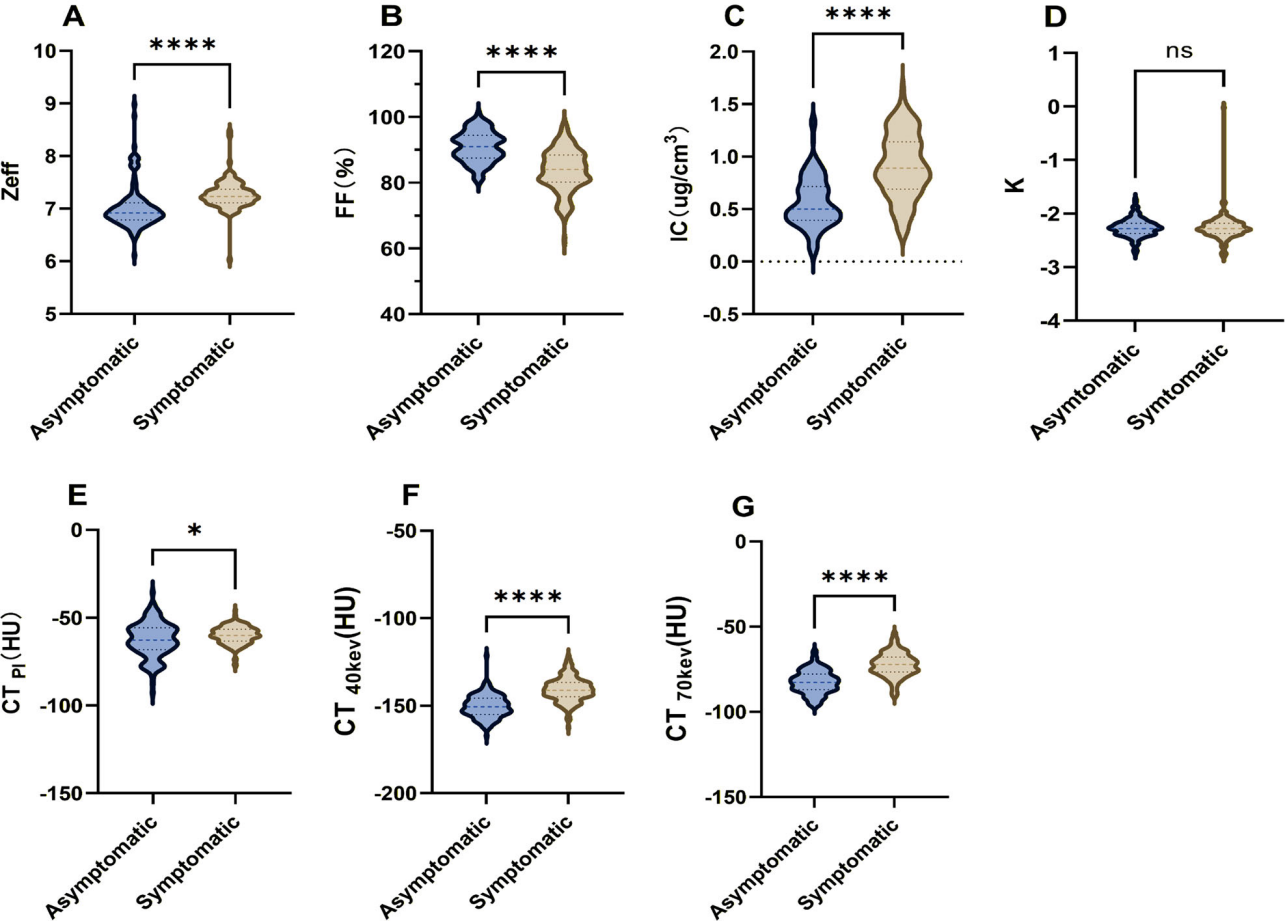
**A**–**G** Comparison of plaque spectral CT parameters between symptomatic and asymptomatic patients. *Z*eff, effective atomic number; FF, fat fraction; IC, iodine concentration; K, the slope of the energy spectrum curve; CT_PI_, attenuation of conventional polyenergetic image; CT_40 keV_, virtual monoenergetic image attenuation at 40 keV; CT_70 keV_, virtual monoenergetic image attenuation at 70 keV. Significance levels are: *p < 0.05; ****p < 0.0001; ns not significant

Compared with the asymptomatic group, symptomatic patients showed greater luminal stenosis (*p* = 0.027) and higher plaque burden (*p* < 0.001). Plaque length, fibrous fatty volume, and necrotic core volume were higher in symptomatic plaques, whereas fibrous volume was lower (all *p* < 0.001). Plaque thickness and calcium volume did not differ significantly between groups (both *p* > 0.05). For PVAT spectral CT parameters, symptomatic patients had higher *Z*eff, IC, CT_PI_, CT_40 keV_, and CT_70 keV_, with a lower FF (all *p* ≤ 0.05). The spectral slope K showed no significant between-group difference (*p* = 0.658) (Table 2 and Fig. 3). The interobserver agreement for assessing plaque characteristics and PVAT parameters, as reflected by the ICC values, varied from 0.758 to 0.865, signifying substantial to excellent consistency between observers. These results are presented in Table S1.

**Table 2:** Analysis of carotid plaque and PVAT parameters in symptomatic and asymptomatic groups

Variables	Asymptomatic (*n* = 137)	Symptomatic (*n* = 169)	*Z*	*p*
Degree of stenosis (%)	53.31 (44.98,66.06)	70.23 (58.88, 79.28)	−2.23	0.027
Plaque burden (%)	60.86 (53.65, 66.50)	63.59 (59.01, 68.83)	−3.17	0.002
Plaque thickness (mm)	3.60 (3.03, 5.09)	3.58 (2.93, 4.86)	−0.43	0.656
Plaque length (mm)	19.64 (19.45, 20.11)	21.85 (21.68, 22.44)	−15.04	0.000
Fibrous volume (mm^3^)	149.71 (118.69, 169.92)	105.43 (76.89, 135.70)	−8.11	0.000
Fibrous fatty volume (mm^3^)	51.81 (26.33, 73.69)	84.03 (57.04, 108.96)	−7.09	0.000
Necrotic core volume (mm^3^)	150.69 (123.61, 180.25)	198.62 (166.65, 221.15)	−8.10	0.000
Calcium volume (mm^3^)	31.68 (28.07, 37.10)	33.00 (26.85, 40.24)	−0.21	0.828
*Z*eff	6.92 (6.79, 7.11)	7.25 (7.13, 7.36)	−9.19	0.000
FF (%)	90.93 (87.50, 94.32)	84.02 (80.18, 88.32)	−9.61	0.000
IC (μg/cm^3^)	0.52 (0.40,0.73)	0.79(0.55, 0.99)	−5.99	0.000
CT_PI_ (HU)	−62.72 (−68.19, −55.81)	−60.10 (−63.32, −56.65)	14.79	0.012
CT_40keV_ (HU)	−150.54 (−154.90, −145.72)	−141.21 (−144.79, −136.75)	−10.73	0.000
CT_70keV_ (HU)	−82.72 (−86.91,−77.82)	−72.74 (−76.51,−67.81)	−10.51	0.000
*K*	−2.67 (−2.79, −2.52)	−2.34 (−2.47, −2.25)	−0.443	0.658

*Zeff* effective atomic number, *FF* fat fraction, *IC* iodine concentration, *CT*_40keV_ virtual monoenergetic image attenuation at 40keV, *CT*_70keV_, virtual monoenergetic image attenuation at 70 keV, *CT*_PI_ attenuations of conventional polyenergetic image, *K* the slope of the energy spectrum curve

### Subgroup analysis

To further elucidate the association between PVAT parameters and symptomatic plaques at different stenosis levels, patients were stratified into mild/moderate and severe extracranial carotid stenosis subgroups according to the NASCET criteria (Fig. S1). In the mild/moderate stenosis subgroup, symptomatic plaques exhibited significantly higher Zeff and IC compared to asymptomatic plaques, along with significantly reduced FF. Additionally, CT_40keV_ and CT_70keV_ were elevated (all *p* < 0.001), and CT_PI_ was higher (*p* = 0.017). In the severe stenosis subgroup, symptomatic plaques showed increased *Z*eff and significantly reduced FF, but no significant differences in IC or CT_PI_ (*p* > 0.05). *K* values did not differ significantly between subgroups (both *p* > 0.05).

### Correlation between carotid plaque characteristics and PVAT spectral CT parameters

**Fig. 4:**
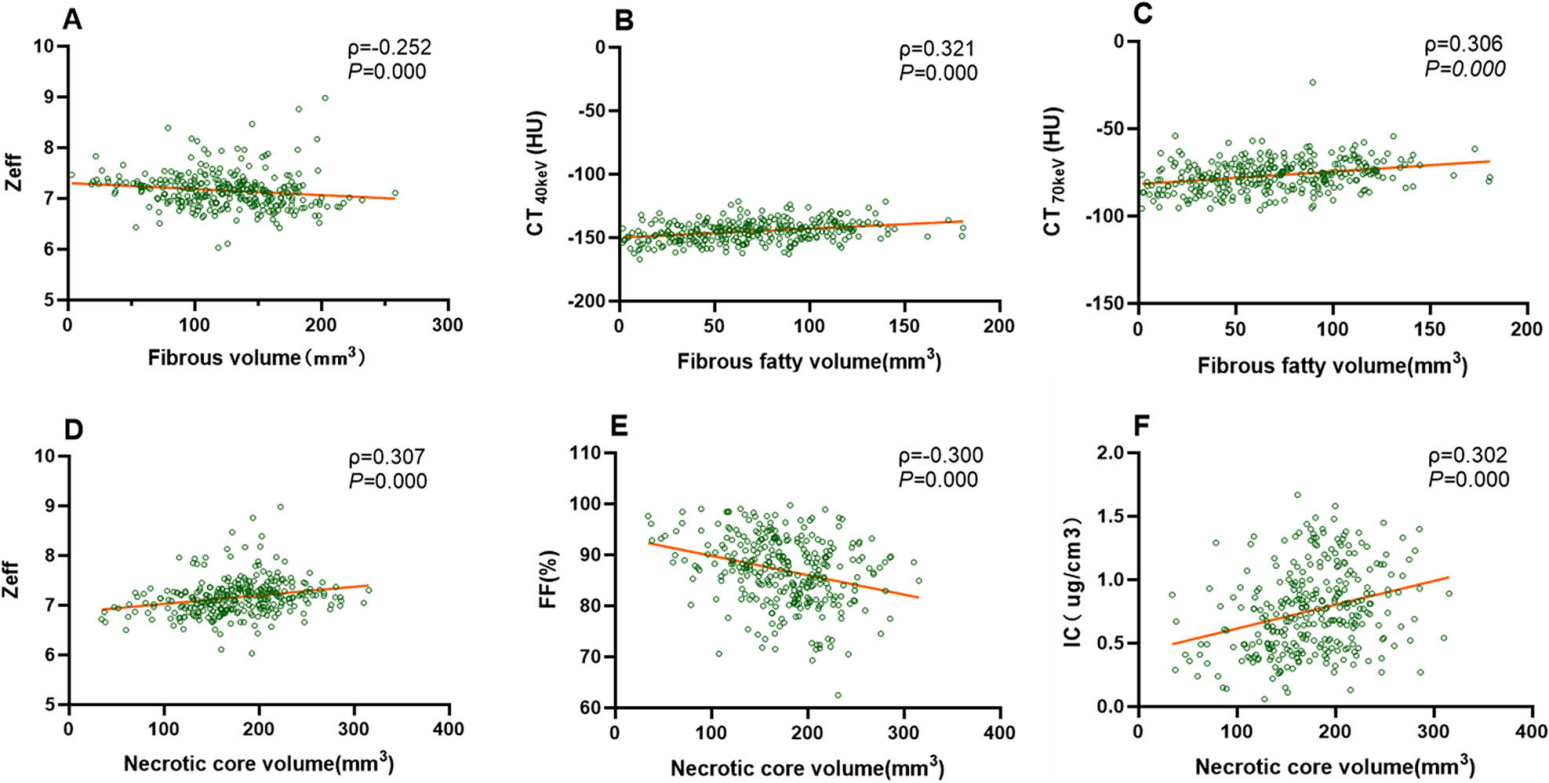
Scatter plots illustrating the relationships between plaque components and spectral CT parameters. Correlation between fibrous volume and *Z*eff (**A**), fibrous fatty volume and CT_40keV_ (**B**) and CT_70keV_ (**C**), necrotic core volume and *Z*eff (**D**), FF (**E**), and IC (**F**). Panels were selected for visualization as representative correlations with the largest absolute Spearman’s ρ among statistically significant pairs; complete correlation results are provided in Table 3. *Z*eff, effective atomic number; FF, fat fraction; IC, iodine concentration; CT_40 keV_, virtual monoenergetic image attenuation at 40 keV; CT_70 keV_, virtual monoenergetic image attenuation at 70 keV

Spearman correlation analysis revealed significant associations between plaque compositional volumes and spectral CT parameters of PVAT (Table 3). Fibrous volume was negatively correlated with *Z*eff, CT_40 keV_, CT_70 keV_ (ρ = −0.252, −0.230, −0.249, respectively; all *p* < 0.001). Fibrous fatty volume was positively correlated with *Z*eff, IC, CT_40 keV_, CT_70 keV_ (ρ = 0.246, 0.277, 0.321, 0.306, respectively; all *p* < 0.001). Necrotic core volume was positively correlated with *Z*eff, IC, CT_40 keV_, CT_70 keV_ (ρ = 0.307, 0.302, 0.299, 0.286, respectively; all *p* < 0.001), and negatively correlated with FF (ρ = −0.300, *p* < 0.001). Calcium volume showed no statistically significant correlations with any of the PVAT spectral CT parameters (all *p* > 0.05) (Fig. 4).

**Table 3:** Correlation between carotid plaque composition and PVAT spectral CT parameters

Variables	ρ^a^	*p*^a^	ρ^b^	*p*^b^	ρ^c^	*P*^c^	ρ^d^	*p*^d^	ρ^e^	*p*^e^	ρ^f^	*p*^f^
Fibrous volume	−0.252	0.000	0.190	0.000	−0.169	0.003	−0.177	0.002	−0.230	0.000	−0.249	0.000
Fibrous fatty volume	0.246	0.000	−0.174	0.002	0.277	0.000	−0.011	0.847	0.321	0.000	0.306	0.000
Necrotic core volume	0.307	0.000	−0.300	0.000	0.302	0.000	0.062	0.283	0.299	0.000	0.286	0.000
Calcium volume	0.057	0.314	0.034	0.556	−0.087	0.128	0.025	0.669	−0.020	0.732	0.016	0.776

*Zeff* effective atomic number, *FF* fat fraction, *IC* iodine concentration, *CT*_*PI*_ attenuations of conventional polyenergetic image, *CT*_40keV_ virtual monoenergetic image attenuation at 40 keV, *CT*_70keV_ virtual monoenergetic image attenuation at 70 keV

^a^ *Z*eff

^b^ FF

^c^ IC

^d^ CT_PI_

^e^ CT_40keV_

^f^ CT_70keV_

### Multivariate logistic regression analysis

Univariate logistic regression analysis showed that plaque burden, fibrous volume, fibrous fatty volume, necrotic core volume, *Z*eff, FF, IC, CT_40 keV_, and CT_70 keV_ were associated with symptomatic plaques (all *p* < 0.001), and CT_PI_ also reached statistical significance (*p* = 0.004). In the multivariate logistic regression model (adjusted for age, sex, hypertension, coronary heart disease, diabetes, hyperlipidemia, smoking history, alcohol drinking history, and medication history), fibrous volume, fibrous fatty volume, necrotic core volume, *Z*eff, FF, IC, and CT_70 keV_ remained independently associated with symptomatic plaques (all *p* < 0.05) (Table 4). Bootstrap internal validation with 1000 resamples demonstrated that the main associations were generally stable across resamples.

**Table 4:** Multivariate logistic regression analysis for predicting symptomatic carotid plaques

Demographic characteristics	Univariate logistic regression		Multivariate logistic regression	
	OR (95% CI)	*p*	OR(95%CI)	*p*
Degree of stenosis	1.025 (0.943−1.115)	0.557		
Plaque thickness (mm)	1.181 (0.233−5.990)	0.840		
Plaque length (mm)	1.334 (0.775−2.296)	0.298		
Plaque Burden (%)	1.048 (1.020−1.076)	0.001	1.049 (0.974−1.128)	0.206
Fibrous volume (mm^3^)	0.973 (0.966−0.980)	0.000	0.948 (0.928−0.968)	0.000
Fibrous fatty volume (mm^3^)	1.026 (1.018−1.033)	0.000	1.020 (1.002−1.038)	0.029
Necrotic core volume (mm^3^)	1.024 (1.017−1.031)	0.000	1.014 (1.014−1.047)	0.000
Calcium volume (mm^3^)	1.005 (0.985−1.025)	0.654		
*Z*eff	12.997 (5.287−31.950)	0.000	5.036 (1.119−22.659)	0.035
FF (%)	0.784 (0.739−0.831)	0.000	0.870 (0.672−0.870)	0.000
IC (0.1 μg/cm^3^)	1.285 (1.177−1.403)	0.000	1.145 (1.147−1.746)	0.001
CT_PI_ (HU)	1.047 (1.015−1.080)	0.004	1.050 (0.961−1.149)	0.282
CT_40keV_ (HU)	1.251 (1.188−1.318)	0.000	1.104 (0.973−1.253)	0.124
CT_70keV_ (HU)	1.233 (1.175−1.294)	0.000	1.173 (1.032−1.333)	0.015
*K*	0.935 (0.320−2.728)	0.902		

*Zeff* effective atomic number, *FF* fat fraction, *IC* iodine concentration, *CT*_PI_ attenuation of conventional polyenergetic image, *CT*_40keV_ virtual monoenergetic image attenuation at 40 keV, *CT*_70keV_ virtual monoenergetic image attenuation at 70 keV, *K* the slope of the energy spectrum curve, *IC* values were rescaled by a factor of 10 to facilitate interpretation of the regression coefficients

Multicollinearity was evaluated by calculating variance inflation factors (VIFs) for all covariates. No substantial multicollinearity was observed (all VIFs < 5)

### The predictive performance of PVAT spectral parameters and plaque quantitative parameters for symptomatic plaques

**Fig. 5:**
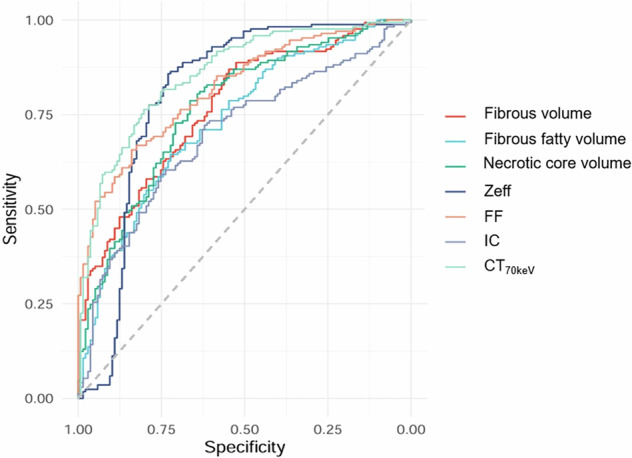
ROC curves of plaque composition and PVAT spectral CT parameters for discriminating symptomatic carotid plaques. *Z*eff, effective atomic number; FF, fat fraction; IC, iodine concentration; CT_70 keV_, virtual monoenergetic image attenuation at 70 keV

Table S2 summarizes the predictive performance of plaque characteristics and PVAT spectral parameters for identifying symptomatic plaques. Among plaque quantitative parameters, necrotic core volume, fibrous volume, and fibrous fatty volume yielded AUCs of 0.769 (95% CI: 0.716–0.822), 0.770 (95% CI: 0.718–0.822), and 0.734 (95% CI: 0.678–0.790), respectively. For PVAT spectral parameters, the AUCs were 0.820 (95% CI: 0.774–0.865) for FF, 0.699 (95% CI: 0.640–0.757) for IC, 0.805 (95% CI: 0.749–0.861) for *Z*eff, and 0.857 (95% CI: 0.815–0.899) for CT_70 keV_ (Fig. 5).

### Incremental discriminatory value of PVAT spectral parameters beyond stenosis severity and plaque composition

To evaluate the incremental discriminatory value of PVAT spectral parameters beyond stenosis severity and plaque composition, we constructed models based on degree of stenosis and plaque composition. A model including degree of stenosis alone (Model A) yielded an AUC of 0.716. After addition of necrotic core volume (Model B), the AUC increased to 0.821. When individual PVAT spectral parameters were further incorporated, the AUC increased to 0.897 for FF (Model C1) and 0.916 for CT_70 keV_ (Model C2). Detailed results are shown in Table 5 and Supplementary Table S3. Compared with Model A, Model B achieved a significantly higher AUC (*p* < 0.001), and further addition of FF (Model C1) or CT_70 keV_ (Model C2) to Model B resulted in additional significant improvements in AUC (both *p* < 0.001).

**Table 5:** Incremental discriminatory value of PVAT spectral parameters beyond degree of stenosis and plaque composition

Model	Variables	AUC	95% CI	Sensitivity	Specificity
A	Degree of stenosis	0.716	0.655–0.773	0.604	0.752
B	Stenosis + necrotic core volume	0.821	0.773–0.865	0.722	0.803
C1	B + FF	0.897	0.859–0.929	0.775	0.891
C2	B + CT_70keV_	0.916	0.884–0.945	0.858	0.839

*AUC* area under the curve, *CI* confidence interval, *PVAT* perivascular adipose tissue, *FF* fat fraction, *CT*_70keV_ virtual monoenergetic image attenuation at 70 keV

Model A was based on the degree of stenosis alone. Model B combined the degree of stenosis with necrotic core volume. Model C models further incorporated individual PVAT spectral parameters

## Discussion

This study analyzed the spectral CT parameters of PVAT and the quantitative characteristics of plaques in patients with carotid atherosclerosis, and further explored the value of these parameters in identifying clinically symptomatic plaques. Our findings emphasize that PVAT spectral CT parameters may serve as potential imaging biomarkers for symptomatic carotid plaques, providing supplementary information regarding microenvironmental changes in perivascular tissues.

As an inflammatory imaging biomarker, PVAT has the core advantage of enabling non-invasive detection [25]. Previous studies have demonstrated that elevated CT attenuation values of PVAT can serve as an indicator of high-risk carotid plaques [9, 11, 15]. Our study found that PVAT attenuation based on energy-spectral CT was significantly elevated in symptomatic carotid plaques, consistent with previous findings [22, 26]. We also observed a reduced FF in PVAT surrounding symptomatic plaques. This reduction may be attributed to adipocyte dysfunction adjacent to diseased plaques, leading to lipid depletion and thereby manifesting as characteristic density changes on CTA [25]. *Z*eff, a spectral metric related to tissue elemental composition, was also higher in PVAT surrounding plaques in symptomatic patients, suggesting potential compositional differences. Similarly, Shinohara et al reported that lipid-rich regions of carotid plaques were associated with lower *Z*eff values [27]. Neovascularization within plaques is closely related to increased plaque activity, leading to an increased risk of inflammation [28]. The increased IC within PVAT may reflect inflammation-driven neovascularization and enhanced vascular permeability. It is worth noting that our study showed no significant difference in PVAT K between the symptomatic and asymptomatic groups, which contradicts previous studies [19]. This negative result may reflect differences in the study population and reconstruction settings. Additionally, calculating *K* values using a relatively narrow low-energy range (40–70 keV) may have reduced the dynamic range of attenuation variation across different energies, thereby limiting intergroup separation. Future studies should further validate the discriminatory value of *K* values under a unified, optimized energy spectrum protocol.

In the stratification analysis based on stenosis severity, PVAT spectral CT parameters could distinguish symptomatic from asymptomatic plaques even within the mild/moderate stenosis range. Conversely, no significant difference in IC values was observed among patients with severe stenosis. Severe carotid stenosis is often accompanied by significant hemodynamic impairment and ipsilateral perfusion deficits [29], which may attenuate or mask perfusion-related PVAT alterations detected in this study. The absence of differences in CT_PI_ suggests that VMIs may more sensitively capture subtle compositional changes within PVAT.

With respect to plaque characteristics, our study confirmed that clinically symptomatic plaques were characterized by larger necrotic cores and reduced fibrous content, consistent with the phenotype that has been tightly linked to ischemic stroke risk in prior MRI and histopathologic studies [30**–**32]. Plaque characteristics are closely associated with the risk of ischemic stroke [33]. Our correlation analysis further elucidated the quantitative relationships between PVAT spectral features and specific plaque components. Higher *Z*eff and IC in PVAT were associated with greater volumes of both fibrous fatty tissue and the necrotic core, while PVAT FF showed an inverse correlation with necrotic core volume. This may be due to the release of inflammatory mediators from necrotic material within the plaque, which activates PVAT, creating a mutually reinforcing vicious cycle [25]. As the necrotic core enlarges, PVAT inflammatory changes become more pronounced, and fat tissue replacement becomes more severe [34]. In addition, this localized inflammation promotes plaque formation, leading to stenosis and increased plaque burden [11]. PVAT may be associated with atherosclerotic plaque phenotype rather than acting as a purely passive bystander [35]. The remodeling process of perivascular fat appears to progress in parallel with the development of luminal and vascular wall lesions, rather than merely coexisting with them [36]. Nevertheless, the results of this study should be interpreted with caution regarding causality. Abnormalities in PVAT may not only contribute to plaque instability through local inflammation and paracrine signaling, but may also represent a secondary component and inflammatory remodeling response of adjacent adipose tissue to plaque activity, vascular inflammation, or ischemic events. Future prospective longitudinal studies combining serial imaging and clinical follow-up are needed to determine whether changes in PVAT precede plaque instability or reflect downstream changes following vascular inflammation or ischemic events.

PVAT spectral parameters demonstrated good diagnostic performance in our study. This suggests that the spectral phenotype of PVAT may be associated with perivascular changes related to clinically symptomatic plaque status, such as perivascular inflammation and metabolic stress, which cannot be fully explained by the degree of lumen stenosis or the internal composition of the plaque. Our findings align with previous dual-energy CT studies indicating that PVAT characteristics correlate with cerebrovascular events and enhance stroke risk prediction beyond conventional imaging markers [19]. By systematically comparing plaque composition volume with multiple PVAT spectral parameters in the same patient cohort, this study provides more concrete evidence of the value of PVAT-related parameters in identifying symptomatic plaques. These findings reinforce the concept that spectral CT-based quantitative assessment of PVAT effectively complements conventional plaque imaging, facilitating more refined risk stratification in patients with carotid atherosclerosis. Importantly, hierarchical modeling showed that adding FF or CT_70 keV_ to the model incorporating the degree of stenosis and necrotic core volume further improved discriminative performance. These findings suggest that PVAT spectral parameters may capture complementary information not fully reflected by conventional luminal or plaque compositional markers alone.

In carotid imaging, spectral CT has been applied for stenosis grading [37] and plaque detection [17]. Even when detailed quantitative analysis of plaque composition is not available, spectral alterations of PVAT may serve as indirect imaging markers of clinically high-risk plaque phenotype, helping to identify high-risk patients with plaques associated with more active perivascular inflammatory changes among those with similar degrees of stenosis [22]. If integrated in future studies with MRI-based plaque composition assessment, radiomics features, and longitudinal stroke outcome follow-up, these metrics may contribute to an integrated risk assessment framework and further improve individualized risk stratification in patients with carotid atherosclerosis.

Our study has some limitations. First, this was a retrospective, single-center study that included only patients who underwent carotid CTA for clinical indications; it did not include an independent external validation cohort. This may introduce selection bias and limit the generalizability of the study’s findings, as well as the representativeness of the study population. Second, plaque classification is based on clinical presentation and brain MRI findings rather than histopathological confirmation; this may introduce uncertainty when determining whether PVAT spectral parameters directly reflect the intrinsic instability of plaques. In addition, this study did not evaluate intraplaque hemorrhage (IPH). Although spectral CT has shown potential in detecting IPH through virtual single-energy imaging or dual-energy ratio, its diagnostic accuracy is still lower than that of MRI. Finally, since the post-processing workstation cannot semi-automatically extract PVAT, manual ROI placement is time-consuming and operator-dependent. The spectral parameters analyzed in this study cannot be obtained from standard single-energy CTA protocols, which currently limits the broader generalizability of these findings.

## Conclusions

Spectral CT-derived PVAT parameters differ between symptomatic and asymptomatic carotid atherosclerosis and are associated with plaque composition, providing complementary noninvasive information for identifying symptomatic plaques.

## Supplementary information

ELECTRONIC SUPPLEMENTARY MATERIAL

## Data Availability

All data generated or analysed during this study are included in this publication.

## Abbreviations

AUC: Area under the curve
CT: Computed tomography
CTA: Computed tomography angiography
FF: Fat fraction
HU: Hounsfield unit
IC: Iodine concentration
K: The slope of the energy spectrum curve
MRI: Magnetic resonance imaging
NASCET: North American Symptomatic Carotid Endarterectomy Trial
PVAT: Perivascular adipose tissue
ROC: Receiver operating characteristic
ROI: Region of interest
TIA: Transient ischemic attack
*Z*eff: Effective atomic number
CT_PI_: Attenuations of conventional polyenergetic image
CT_40keV_: Virtual monoenergetic image attenuation at 40 keV
CT_70keV_: Virtual monoenergetic image attenuation at 70 keV
