# Supplementary material for: Spectral CT parameters of perivascular adipose tissue as non-invasive biomarkers for identifying symptomatic carotid atherosclerosis
Source: Insights Imaging. 2026 Jul 22;17:190. doi: 10.1186/s13244-026-02354-w (PMC13391981; doi:10.1186/s13244-026-02354-w)
Supplement: Supplementary file 1 — ELECTRONIC SUPPLEMENTARY MATERIAL [file 13244_2026_2354_MOESM1_ESM.pdf]

Spectral CT Parameters of Perivascular Adipose Tissue as Non-Invasive Biomarkers for Identifying Symptomatic Carotid Atherosclerosis

ELECTRONIC SUPPLEMENTARY MATERIAL

Fig S1. (A-G) Comparison of perivascular adipose tissue (PVAT) spectral CT parameters between symptomatic and asymptomatic plaques across mild/moderate and severe stenosis subgroups.

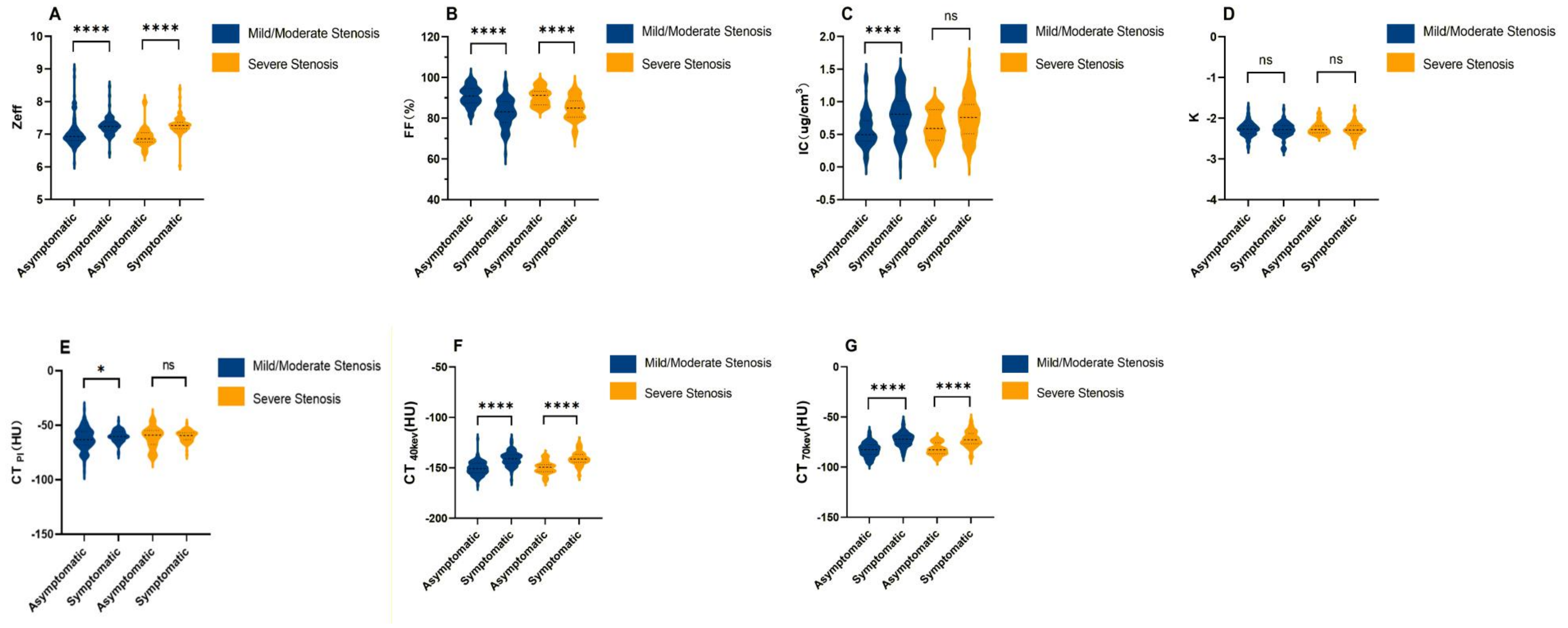

Note: Zeff, effective atomic number; FF, fat fraction; IC, iodine concentration; K, the slope of the energy spectrum curve; CT_{PI}, attenuations of conventional polyenergetic image; $CT_{40\ keV}$, virtual monoenergetic image at 40 keV; $CT_{70\ keV}$, virtual monoenergetic image attenuation at 70 keV.

Significance levels are: *P < 0.05; ***P < 0.001; ns: not significant.

Table S1. The ICC for evaluating plaque features and PVAT parameters.

Variables	ICC	95% CI	P
Degree of stenosis	0.766	0.733, 0.799	< 0.001
Plaque thickness (mm)	0.771	0.738, 0.803	< 0.001
Plaque length (mm)	0.802	0.774, 0.830	< 0.001
Zeff	0.845	0.822, 0.868	< 0.001
FF (%)	0.789	0.759, 0.819	< 0.001
IC (μg/cm^3)	0.784	0.753, 0.814	< 0.001
CT_{40KeV}	0.785	0.754, 0.816	< 0.001
CT_{70keV}	0.758	0.724, 0.792	< 0.001
CT_{PI}	0.865	0.845, 0.885	< 0.001
K	0.783	0.752, 0.814	< 0.001
Plaque burden (%)	0.782	0.751, 0.813	< 0.001
Fibrous volume(mm^3)	0.764	0.731, 0.797	< 0.001
Fibrous fatty volume(mm^3)	0.829	0.804, 0.854	< 0.001
Necrotic core volume(mm^3)	0.827	0.802, 0.852	< 0.001
Calcium volume(mm^3)	0.831	0.806, 0.855	< 0.001

Note: ICC, intraclass correlation coefficient; CI, confidence interval; Zeff, effective atomic number; FF, fat fraction; IC, iodine concentration; $CT_{40\ keV}$, virtual monoenergetic image attenuation at 40 keV; $CT_{70\ keV}$, virtual monoenergetic image attenuation at 70 keV; CT_{PI}, attenuations of conventional polyenergetic image; K, the slope of the energy spectrum curve.

ICC values were derived from the analysis of repeated measurements conducted by two reviewers (X.L. and J.S.) with substantial experience in cardiovascular imaging. For each variable, ICC was calculated, and the 95% confidence intervals (CIs) were reported to

determine the degree of agreement between the measurements. ICCs were calculated for the interobserver agreement: ICC < 0.40, poor; 0.41–0.60, moderate; 0.61–0.80, substantial; and > 0.80, excellent.

Table S2. The predictive performance of PVAT spectral parameters and plaque quantitative parameters for symptomatic plaques.

	AUC (95%CI)	Sensitivity (95%CI)	Specificity (95%CI)	PPV (95%CI)	NPV (95%CI)
FF	0.820 (0.774–0.865)	0.164 (0.102 – 0.216)	0.342 (0.272 – 0.408)	0.174 (0.09 – 0.232)	0.343 (0.264 – 0.410)
IC	0.699 (0.640–0.757)	0.751(0.683 – 0.821)	0.587 (0.520 – 0.665)	0.600 (0.531 – 0.670)	0.752 (0.670 – 0.823)
Zeff	0.805 (0.749–0.861)	0.863 (0.801 – 0.899)	0.728 (0.650-0.803)	0.801 (0.728-0.854)	0.811 (0.726-0.874)
$CT_{70\ keV}$	0.857 (0.815–0.899)	0.859 (0.804-0.908)	0.724 (0.644-0.802)	0.791 (0.725-0.851)	0.809 (0.732-0.879)
fibrous volume	0.770 (0.718–0.822)	0.446 (0.371 – 0.539)	0.131 (0.082 – 0.184)	0.301 (0.228 – 0.357)	0.231 (0.140 – 0.312)
fibrous fatty volume	0.734 (0.678–0.790)	0.721 (0.648 – 0.802)	0.644 (0.568 – 0.719)	0.623 (0.554 – 0.704)	0.744 (0.671 – 0.805)
necrotic core volume	0.769 (0.716–0.822)	0.662 (0.587 – 0.742)	0.785 (0.729 – 0.849)	0.715 (0.641 – 0.789)	0.739 (0.684 – 0.813)

Note: AUC, area under the curve; PPV, positive predictive value; NPV, negative predictive value; FF, fat fraction; IC, iodine concentration; Zeff, effective atomic number; $CT_{70\ keV}$, virtual monoenergetic image attenuation at 70 keV.

Table S3. Discriminative performance of stenosis severity, plaque compositional variables, and PVAT spectral parameters

Model group	Model	Variables	AUC	95% CI	Sensitivity	Specificity
A	A	Degree of stenosis	0.716	0.655, 0.773	0.604	0.752
B	B1	A + plaque burden	0.735	0.676, 0.789	0.698	0.715
B	B2	A + fibrous volume	0.812	0.765, 0.857	0.722	0.781
B	B3	A + fibrous fatty volume	0.794	0.742, 0.843	0.609	0.869
B	B4	A + necrotic core volume	0.821	0.773, 0.865	0.722	0.803
B	B5	A + calcium volume	0.716	0.658, 0.775	0.538	0.818
C	C1	B4 + Zeff	0.847	0.800, 0.888	0.775	0.81
C	C2	B4 + FF	0.897	0.859, 0.929	0.775	0.891
C	C3	B4 + iodine concentration	0.848	0.801, 0.887	0.734	0.781
C	C4	B4 + CT_{70keV}	0.916	0.884, 0.945	0.858	0.839

Note：AUC, area under the receiver operating characteristic curve; CI, confidence interval; PVAT, perivascular adipose tissue; Zeff, effective atomic number; FF, fat fraction, Zeff, effective atomic number; $CT_{70\ keV}$, virtual monoenergetic image attenuation at 70 keV.

Model A included stenosis severity alone. In Model B, individual plaque compositional variables were added one at a time to Model A. In Model C, individual PVAT spectral parameters were further added one at a time to the best-performing Model B, which consisted of stenosis severity and necrotic core volume.
